# Characterization and localization of antigens for serodiagnosis of human paragonimiasis

**DOI:** 10.1007/s00436-020-06990-z

**Published:** 2021-01-08

**Authors:** Kurt C. Curtis, Kerstin Fischer, Young-Jun Choi, Makedonka Mitreva, Gary J. Weil, Peter U. Fischer

**Affiliations:** 1 Division of Infectious Diseases, Department of Medicine, Washington University School of Medicine, 4444 Forest Park Blvd, St. Louis, MO 63110 USA; 2 McDonnell Genome Institute, Washington University School of Medicine, St. Louis, MO 63110 USA

**Keywords:** Immunodiagnosis, Paragonimiasis, *Paragonimus*, Trematode, Recombinant antigens

## Abstract

Paragonimiasis is a foodborne trematode infection that affects 23 million people, mainly in Asia. Lung fluke infections lead frequently to chronic cough with fever and hemoptysis, and are often confused with lung cancer or tuberculosis. Paragonimiasis can be efficiently treated with praziquantel, but diagnosis is often delayed, and patients are frequently treated for other conditions. To improve diagnosis, we selected five *Paragonimus kellicotti* proteins based on transcriptional abundance, recognition by patient sera, and conservation among trematodes and expressed them as His-fusion proteins in *Escherichia coli*. Sequences for these proteins have 76–99% identity with amino acid sequences for orthologs in the genomes of *Paragonimus westermani*, *Paragonimus heterotremus*, and *Paragonimus miyazakii*. Immunohistology studies showed that antibodies raised to four recombinant proteins bound to the tegument of adult *P. kellicotti* worms, at the parasite host interface. Only a known egg antigen was absent from the tegument but present in developing and mature eggs. We evaluated the diagnostic potential of these antigens by Western blot with sera from patients with paragonimiasis (from MO and the Philippines), fascioliasis, and schistosomiasis, and with sera from healthy North American controls. Two recombinant proteins (a cysteine protease and a myoglobin) showed the highest sensitivity and specificity as diagnostic antigens, and they detected antibodies in sera from paragonimiasis patients with early or mature infections. In contrast, antibodies to egg yolk ferritin appeared to be specific marker for patients with adult fluke infections that produce eggs. Our study has identified and localized antigens that are promising for serodiagnosis of human paragonimiasis.

**Supplementary Information:**

The online version contains supplementary material available at 10.1007/s00436-020-06990-z.

## Introduction

Foodborne trematode (FBT) infections are important neglected tropical diseases (NTDs) that affect about 56 million people and cause significant morbidity (Furst et al. 2012). Among FBT infections, lung flukes of the genus *Paragonimus* are arguably the most important group with an estimated 23 million human infections (Keiser and Utzinger 2009). *Paragonimus* species are widely distributed in animals in Asia, Africa, and the Americas, but the infection risk for humans depends largely on whether humans ingest raw or undercooked crustaceans that act as intermediate hosts. For *Paragonimus westermani*, ingestion of raw meat from mammalian paratenic hosts provides an additional route of infection (Blair 2014). Human *Paragonimus* infection can be efficiently treated with a short course of praziquantel, but diagnosis is challenging, because infected people are often misdiagnosed and treated for pneumonia, tuberculosis, or cancer (Fischer and Weil 2015).

*Paragonimus kellicotti* is the only *Paragonimus* species endemic in the USA. Human *P. kellicotti* infections are rare, but in recent years, a number of serious cases have been reported from MO and adjacent states (Bahr et al. 2017; Lane et al. 2012). While the symptoms usually start 2 to 16 weeks after crayfish ingestion, accurate diagnosis is often delayed for a period of weeks to many months after the onset of symptoms (Lane et al. 2012). In order to improve the serological diagnosis of *P. kellicotti* infection, we developed a small animal infection model to provide adult worms for antigen production. The native adult worm antigen was used to develop a Western blot assay that was sensitive and specific for paragonimiasis (Fischer et al. 2011b, 2013). Unfortunately, the native parasite antigen is not widely available, and recombinant antigen-based antibody tests are preferable in terms of feasibility and reproducibility. Therefore, we used a systems biology approach that included transcriptome sequencing followed by assembly and annotation, immunoprecipitation using patients’ sera, and proteomics to identify candidate antigens for serodiagnosis of paragonimiasis (McNulty et al. 2014). In this study, we build on our progress by further characterizing five of these antigens and evaluate their potential value for diagnosis of *P. kellicotti* and *P. westermani* infections. These included a cysteine protease (Pk00394_txpt2; MK050848), a cystatin (Pk45107-txpt2; MK050849), a myoglobin (Pk34178-txpt1; MK050847), a microbial defense protein (Pk39524_txpt1; MK050850), and an egg yolk ferritin (Pk48313-txpt; MK050851) (McNulty et al. 2014). Four of these serodiagnostic antigen candidates were among the top 25 immunoreactive adult *P. kellicotti* proteins, show relatively little conservation in other helminth genera, and represent distinct protein families (McNulty et al. 2014). The egg yolk ferritin was a highly abundant protein in adult worm extract and was target of a previous immunoassay for *P. westermani* (Kim et al. 2002a, b). So far, only few *Paragonimus* proteins have been recombinantly expressed and evaluated for their serodiagnostic potential. Among these are, apart from the egg yolk ferritin, different cysteine proteases of *P. westermani*, *Paragonimus pseudoheterotremus*, and *Paragonimus skrjabini* (Kim et al. 2000; Park et al. 2001; Yang et al. 2004; Yoonuan et al. 2016; Yu et al. 2017). *Paragonimus* species contain an expanded number of cysteine protease family members, and many of them are secreted and highly immunogenic, making them promising diagnostic antigens (Blair et al. 2016).

We cloned and expressed the proteins in *Escherichia coli*, purified the recombinant proteins, and tested their serodiagnostic potential with sera from patients infected with *P. kellicotti* or *P. westermani*. As negative controls, we used sera of patients infected with or without other trematode infections. In order to better characterize these target protein, we produced polyclonal mouse antibodies using recombinant protein and localized the antigens in adult *P. kellicotti* flukes.

## Material and Methods

### Parasite material

Adult stage *P. kellicotti* parasites were obtained as described previously from experimentally infected Mongolian gerbils, *Meriones unguiculatus* (Fischer et al. 2011b). The use of gerbils for *P. kellicotti* infections was approved by the Washington University Animal Studies Committee and followed the IACUC guidelines. Adult worms (45 days p.i.) were fixed in 4% buffered formalin for immunohistology studies or stored at − 80 °C for RNA extraction.

### Patient sera

The study protocol was approved by the Human Research Protection Office at Washington University School of Medicine. This study included samples that were tested in our previous Western blot study with native *P. kellicotti* adult worm antigen (Fischer et al. 2013). Samples from patients with proven *P. kellicotti* infection were exclusively from MO, while samples with proven *P. westermani* infection (kindly provided by Dr. P. Wilkinson at the US Centers for Disease Control and Prevention) were from the Philippines.

### Protein expression and purification

Total RNA was isolated from six adult *P. kellicotti* using the PureLink RNA mini kit (Thermo Fisher Scientific, Waltham MA, USA), and cDNA was synthesized using the 1 Script kit (BioRad, Hercules, CA, USA) according to the manufacturer’s instructions. Diagnostic candidates were amplified with 50 ng of cDNA template and Platinum Pfx DNA polymerase (Thermo Fisher Scientific), and 20 pmol of primers (Supplemental Table S1). PCR products were Sanger sequenced and sequences of interest were submitted to GenBank

### Antigen sequence analysis

The Sanger sequencing of the cDNA clones and the subsequent translation of the open reading frame generated protein sequences for comparative analysis (AZZ10059–AZZ10063). These sequences were used to identify orthologous sequences in other *Paragonimus* species (Rosa et al. 2020). The closest orthologs for *P. kellicotti* antigens in other *Paragonimus* species were retrieved by BLASTP against complete deduced proteomes that were generated by the *Paragonimus* genome sequencing project (Martin et al. 2015). Sequences are available at GenBank under BioProject id PRJNA284523 (*P. heterotremus*), PRJNA245325 (*P. miyazakii*), and PRJNA219632 (*P. westermani*). In addition, we ran a blast search of the *P. kellicotti* Cp-6 and Myo-1 against the orthologous groups from other FBTs and identified the top BLASTP hit. For *Opisthorchis viverrini*, no significant hit was obtained for the orthologous groups and we ran BLASTP against the GenBank NR database.

### Production and purification of recombinant *P. kellicotti* proteins

PCR products were ligated into the directional pET100/D-TOPO His-tagged expression vector (Invitrogen, Carlsbad, CA, USA) according to the manufacturer’s instructions, and proteins were expressed using BL21 (DE3) *E. coli* cells (Sigma, St. Louis, MO, USA). Recombinant proteins were purified with His-Select Cobalt Affinity Get h8162 resin (Sigma).

### Western blot

Western blot antibody assays were performed as previously described with 10 μg of antigen protein per centimeter of nitrocellulose (NC) membrane. Test samples included sera from individuals infected with *P. kellicotti*, *P. westermani*, *Schistosoma mansoni*, or other helminths, and with sera from uninfected North Americans (Fischer et al. 2013). Individual 3 mm nitrocellulose (Invitrogen) strips were incubated with primary antibodies in mouse or human sera diluted 1:100 in PBS/Tween for 2 h. Strips were washed in PBS/Tween three times and incubated in alkaline phosphatase-conjugated anti-IgG or anti-IgG4 secondary antibodies (SouthernBiotech, Birmingham, AL, USA) diluted in PBS/Tween for 1 h at room temperature. After washing, antibody binding was revealed by incubating the NC strips in NBT/BCIP (Fischer et al. 2013).

### Production of antibodies to recombinant proteins

Polyclonal antibodies were raised in BALB/c mice by s.c. immunization with 20 μg of recombinant proteins (rCP-6, rMYO-1, rCYS-2, rMDP, and rEYF) in complete Freund’s adjuvant (1st immunization) or in incomplete Freund’s adjuvant (boosting injections). Sera were stored at − 20 °C until use. These antibodies served as positive controls for Western blot assays to verify that target antigens were present on NC membranes.

### Immunolocalization of recombinant antigens

Formalin-fixed, adult *P. kellicotti* flukes were embedded in paraffin and sectioned at 5 μm. Sections were stained with hematoxylin and eosin to delineate general morphology. Two blocks with three flukes per block were sectioned, stained, and analyzed. The alkaline phosphatase-anti-alkaline phosphatase (APAAP) technique was used as previously described for immunostaining (Fischer et al. 2011a, 2017). Briefly, the unstained tissue sections were rehydrated and incubated in 10% bovine serum albumin (Sigma) for 30 min to reduce background staining. Sections were then incubated with murine antibodies to *P. kellicotti* proteins at dilutions of 1:50 to 1:200 in phosphate-buffered saline containing 0.1% Triton-X and 0.1% bovine serum albumin. A dilution of 1:100 of the primary antibodies resulted in the best signal to noise ratio and was used for all further immunostainings. Polyclonal rabbit anti-mouse IgG (Dako, Carpinteria CA, USA) was applied as the secondary antibody, followed by incubation with APAAP (1:40, Sigma). The chromogen Fast Red TR salt (Sigma) was used as the substrate, and hematoxylin (Merck, Darmstadt, Germany) served as the counter-stain. The slides were examined with an Olympus-BX40 microscope (Olympus, Tokyo, Japan) and photographed with an Olympus DP70 digital camera.

For fluorescence microscopy, Alexa Fluor 488 anti-mouse IgG (Invitrogen, green fluorescence) was used as the secondary antibody, and wheat germ agglutinin 633 (200 μg/ml, Invitrogen, red fluorescence), and DAPI (Prolong Antifade with DAPI, Molecular Probes by Life Technologies, Carlsbad, CA, USA, blue fluorescence) were used to label membranes and double-stranded DNA, respectively (Fischer et al. 2017; McNulty et al. 2017). Sections were examined with a wide field fluorescence microscope (WFFM, Zeiss Axios Imager Upright Fluorescence Microscope) with plan-apochromat × 100 oil, × 63, or × 40 objectives.

## Results

### Expression of immunoreactive proteins

**Fig. 1:**
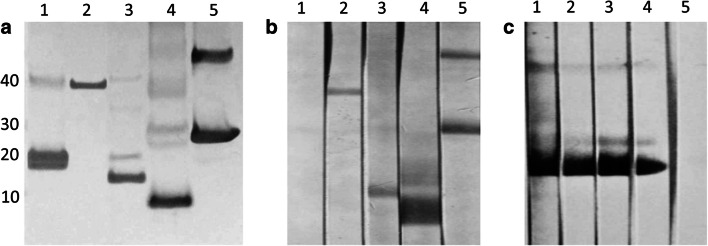
Western blot analysis of recombinant (**a**, **c**) and native antigens (**b**). **A** recombinant antigen was run on a gel, blotted and probed using a monoclonal antibody against the His-tag on the recombinant antigen. The antibody detected the recombinant proteins at the expected size, but sometimes double bands were observed for example due to suboptimal reduction of protein and detection of dimers (lane 5). Lane 1, rMYO-1; lane 2, rCP-6; lane 3, rCYS-2; lane 4, rMDP; lane 5, rEYF. **b** 20 μg of soluble whole worm extract was separated on a gel, blotted and probed using polyclonal mouse antisera, generated against the recombinant protein. With exception of MYO-1, the antibodies recognized a native protein of the expected size. Lane 1, anti-rMYO-1; lane 2, anti-rCP-6; lane 3, anti-rCYS-2; lane 4, anti-rMDP; lane 5, anti-rEYF. **c** rMYO-1 antigen was run on a gel, blotted and probed using polyclonal mouse antiserum raised against rMYO-1 at different dilutions. Lane 1, 1000; lane 2, 1:2000; lane 3, 1:3000; lane 4, 1:4000; lane 5, negative control

cDNAs of five *P. kellicotti* immunoreactive antigens were chosen for expression: a cysteine proteinase (CP-6), a myglobin (MYO-1), a cystatin (CYS-2), a microbial defense protein (MDP), and an egg yolk ferritin (EYF) (Table 1). The recombinant proteins had the expected molecular masses (Fig. 1). However, rMYO-1 showed a more diffuse band, which could be caused by the presence of a second, slightly smaller protein, or partial degradation. The antibody directed against the His-tag recognized rEYF at approximately 24 kDa and a second band at approximately 48 kDa indicative of incomplete reduction and detection of a dimer. When we tested polyclonal antibodies raised against the recombinant proteins by Western blot with crude adult worm extract as antigen, we obtained similar results for CP-6, CYS-2, MDP, and EYF with lower kDa values (consistent with the absence of the His-tag in the native proteins). However, antibodies raised to rMYO-1 did not react with antigens in the worm extract (Fig. 1b), but the antibodies bound to rMYO protein at the expected size (Fig. 1c).

**Table 1:** Summary of the five *P. kellicotti* proteins expressed in *E.coli*. These proteins include cysteine protease-6 (CP-6), cystatin-2 (CYS-2), myoglobin-1 (MYO-1), a microbial defense protein (MDP), and an egg yolk ferritin (EYF). The molecular mass of the recombinant protein includes 4 kDa of the His-tag

Clone designation	Transcript (McNulty et al. 2014)	Top BLASTP	Expected size (kDa)	GenBank acc #
Cp-6	Pk00527	gi|67773374|gb|AAY81944.1| cysteine protease 6 (*Paragonimus westermani*)	40.7	AZZ10060
Cys-2	Pk140546	gi|150404782|gb|ABR68549.1| cystatin-2 (*Clonorchis sinensis*)	16.1	AZZ10061
Myo-1	Pk120808	gi|59895953|gb|AAX11352.1| myoglobin 1 (*Paragonimus westermani*)	20.4	AZZ10059
Mdp	Pk131774	gi|379991184|emb|CCA61804.1| MF6p protein, partial (*Fasciola hepatica*)	12.4	AZZ10062
Eyf	Pk145008	gi|13625997|gb|AAK35224.1|AF367368_1 yolk ferritin (*Paragonimus westermani*)	23.6	AZZ10063

### Conservation of selected immunoreactive proteins

We compared the amino acid sequences of the five selected *P. kellicotti* proteins with orthologous proteins from the *P. westermani*, *P.miyazakii*, *P. heterotremus* genomes (Martin et al. 2018). Close orthologs of the *P. kellicotti* sequences for CP-6, MYO-1, CYS-2, MDP, and EYF were identified in the genomes of all three species with amino acid identities that ranged between 79 and 99% (Table 2). These five proteins were all highly conserved between the four species, and there were only minor differences. MDP was the most highly conserved protein with identities between 96 and 99%, while CP-6 was the least conserved protein with identities between 79 and 86%. Still, this high degree of conservation of the selected serodiagnostic antigen candidates may facilitate the development of a pan-*Paragonimus* serodiagnostic antigen. This is supported by the fact that the selected antigen candidates are conserved not only among Asian, but also North American species.

**Table 2:** Comparison of the amino acid sequences of the five *P. kellicotti* serodiagnostic antigen candidates (CP-6, CYS-2, MYO-1, MDP, EYF) with the closest ortholog in the genomes of *P. westermani* (BioProject PRJNA219632), *P. miyazakii* (BioProject PRJNA245325), and *P. heterotremus* (BioProject PRJNA284523).

	Alistat MSA metrics					Best *P. heterotremus* hit		Best *P. miyazakii* hit		Best *P. westermani* hit	
Description	Align length (aa)	Avg. % id	Most related pair %	Most unrelated pair %	Most distant seq %	Name	BLASTP % id to clone	Name	BLASTP % id to clone	Name	BLASTP % id to clone
Pk120808 (MYO-1)	149	92	95	89	91	PHET_00701	95	EG68_01357	95	P879_04241	90
Pk00527 (CP-6)	325	80	86	76	80	PHET_03802	79	EG68_11450	86	P879_11129	82
Pk140546 (CYS-2)	122	96	98	94	95	PHET_06350	96	EG68_11520	97	P879_09965	96
Pk131774 (MDP)	103	95	99	93	96	PHET_08884	97	EG68_09740	99	P879_03998	96
Pk145008 (EYF)	211	92	94	90	91	PHET_10870	93	EG68_11623	91	P879_00428	90

### Immunolocalization of candidate diagnostic proteins in adult *P. kellicotti*

**Fig. 2:**
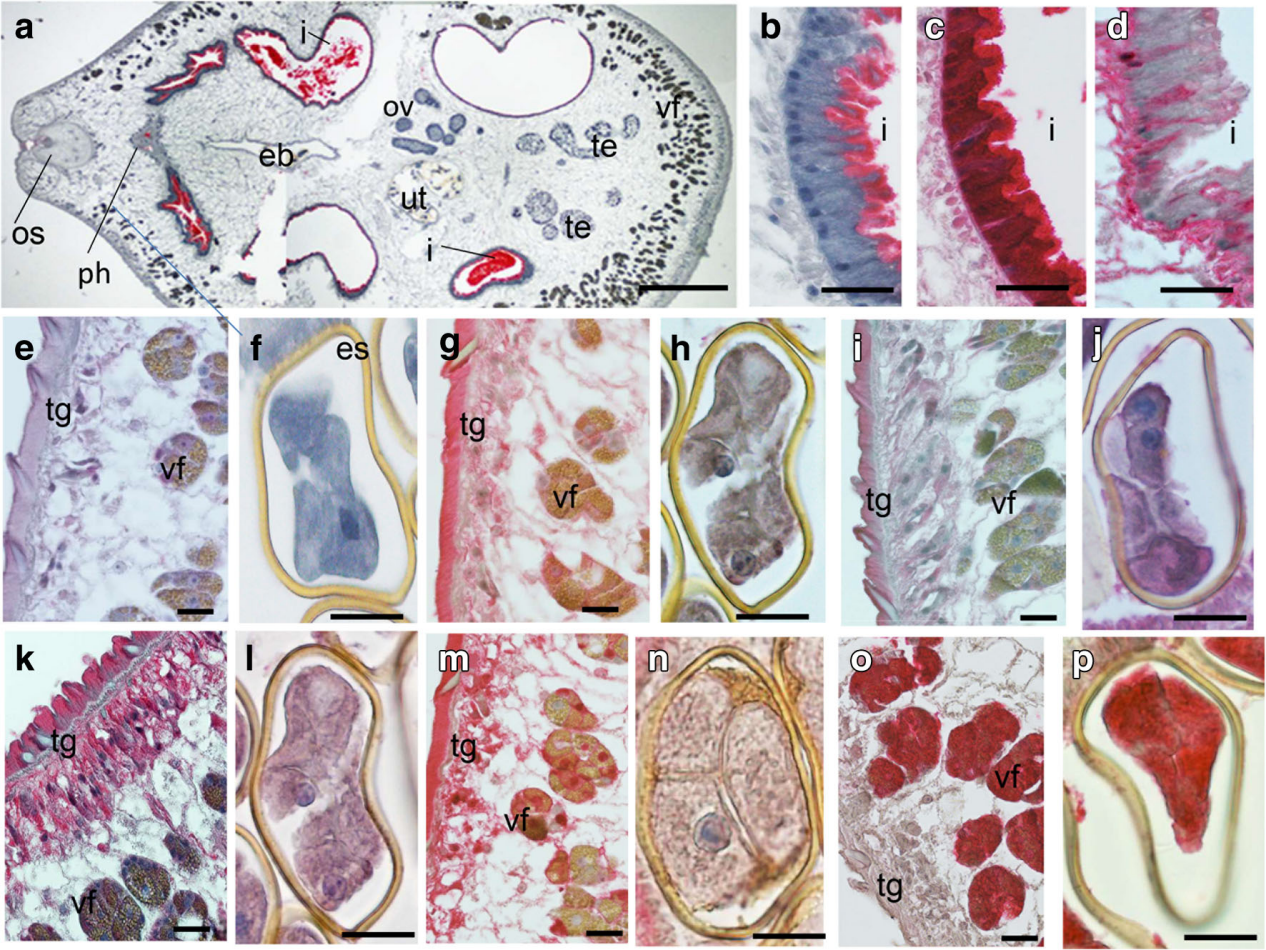
Immunohistological localization of five candidate serodiagnostic antigens in adult *P. kellicotti* adult worms. Panels **a**, **b**, **e**, **f** Negative controls tested with a pre-immune mouse serum. No red staining was detected in any tissue except for the inner lining of the intestine (**a**, **b**). **c**, **g**, **h** Localization of CP-6 using a polyclonal mouse antibody shows intense staining of the intestinal wall (**c**) and the tegument, with weak staining of the vitelline glands (**g**). No staining was observed in the intrauterine eggs (**h**). **i**, **j** Localization of CYS-2 shows moderate to weak staining of the tegument and vitelline glands (**i**) and the eggs (**j**). **k**, **l** Localization of MYO-1 shows strong staining of the tegument and weaker labeling of the vitelline gland (**k**) and the eggs (**l**). **d**, **m**, **n** Localization of MDP shows strong staining of the inner part of the intestinal epithelium (**d**), the tegument and the vitelline glands (**m**) but little staining in the eggs (**n**). **o**, **p** Localization of EYF shows strong labeling limited to the vitelline glands (**o**) and the eggs (**p**). Os, oral sucker; ph, pharynx; eb, excretory bladder; I, intestine; tg, tegument; ut, uterus; te, testis; ov, ovary; vf, vitelline follicle; es, egg shell. Scale bar for **a** is 500 μm and for all others 20 μm

All five antigens showed distinct localization patterns (Fig. 2). Pre-immune murine sera did not label tegument, ova (Fig. 2e, f), or other structures except from a part of the intestine. The inner lining of the intestine of *P. kellicotti* was labeled with all immune and negative control sera. This non-specific staining is probably because of endogenous alkaline phosphatase that is detected by the anti-alkaline phosphatase antibody component in the APAAP localization protocol (Fig. 2a, b). However, the distinct non-specific labeling of parts of the intestine was easily differentiated from specific antibody staining of the intestine (Fig. 2c, d). In agreement with this observation, no unspecific labeling was observed when Alexa Fluor 488 anti-mouse IgG was used as secondary antibody was used that does not contain an anti-alkaline phosphatase antibody like the APAAP complex.

**Fig. 3:**
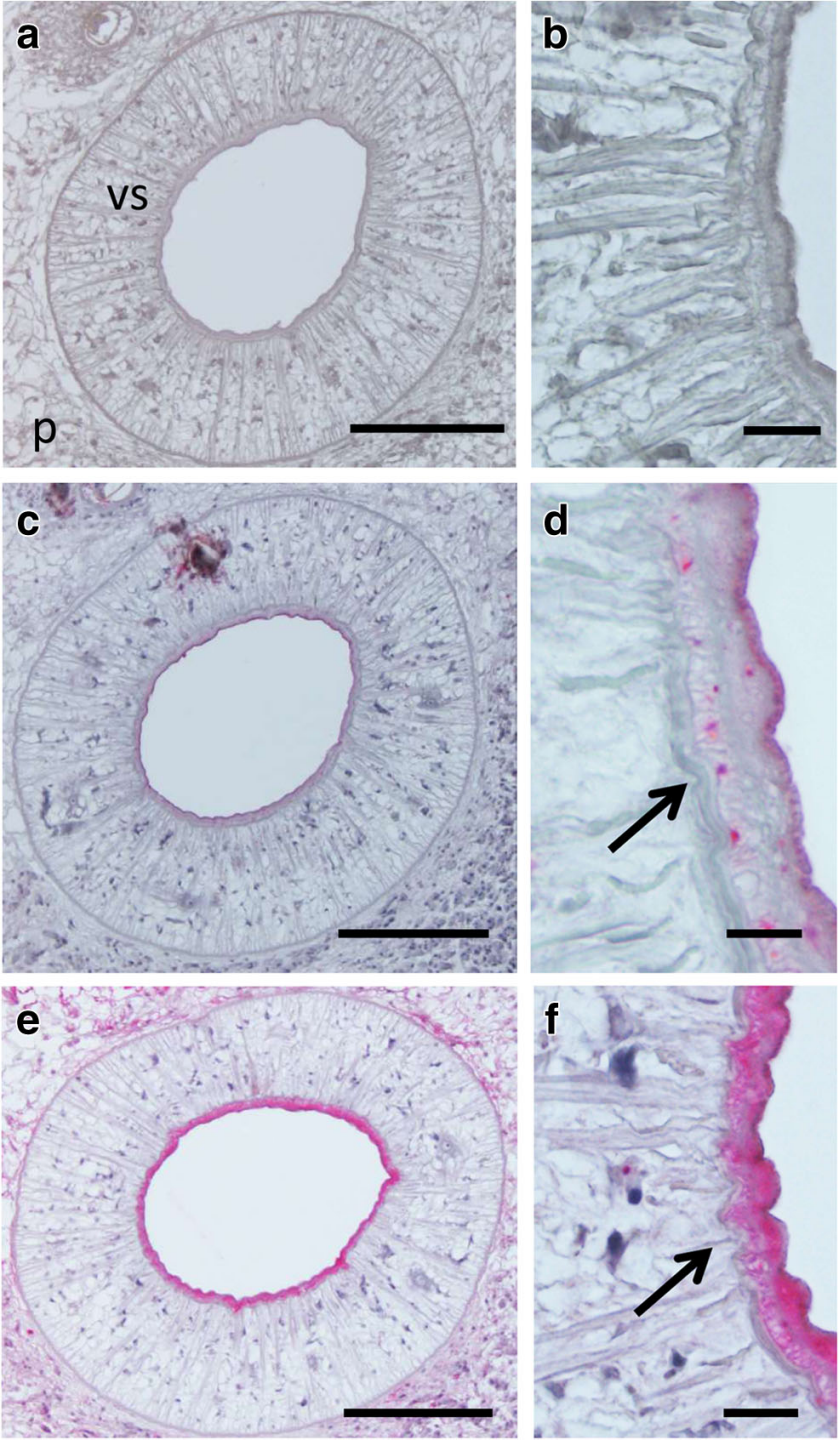
Immunohistological localization of antigens with serodiagnostic potential in the ventral sucker of adult *P. kellicotti* flukes using the APAAP method. **a**, **b** Negative control using a pre-immune serum of a mouse used for antibody generation shows no staining. **c**, **d** Localization of CP-6 shows staining of the outer part of the ventral sucker and some granular staining of single cells (arrow). No staining was observed in the parenchyma. **e**, **f** Localization of MDP shows intense staining of the entire inner part of the ventral sucker and weaker staining in the parenchyma. Vs, ventral sucker; p, parenchyma. Scale bar for A, C, E is 100 μm and for B, D, E 10 μm

**Fig. 4:**
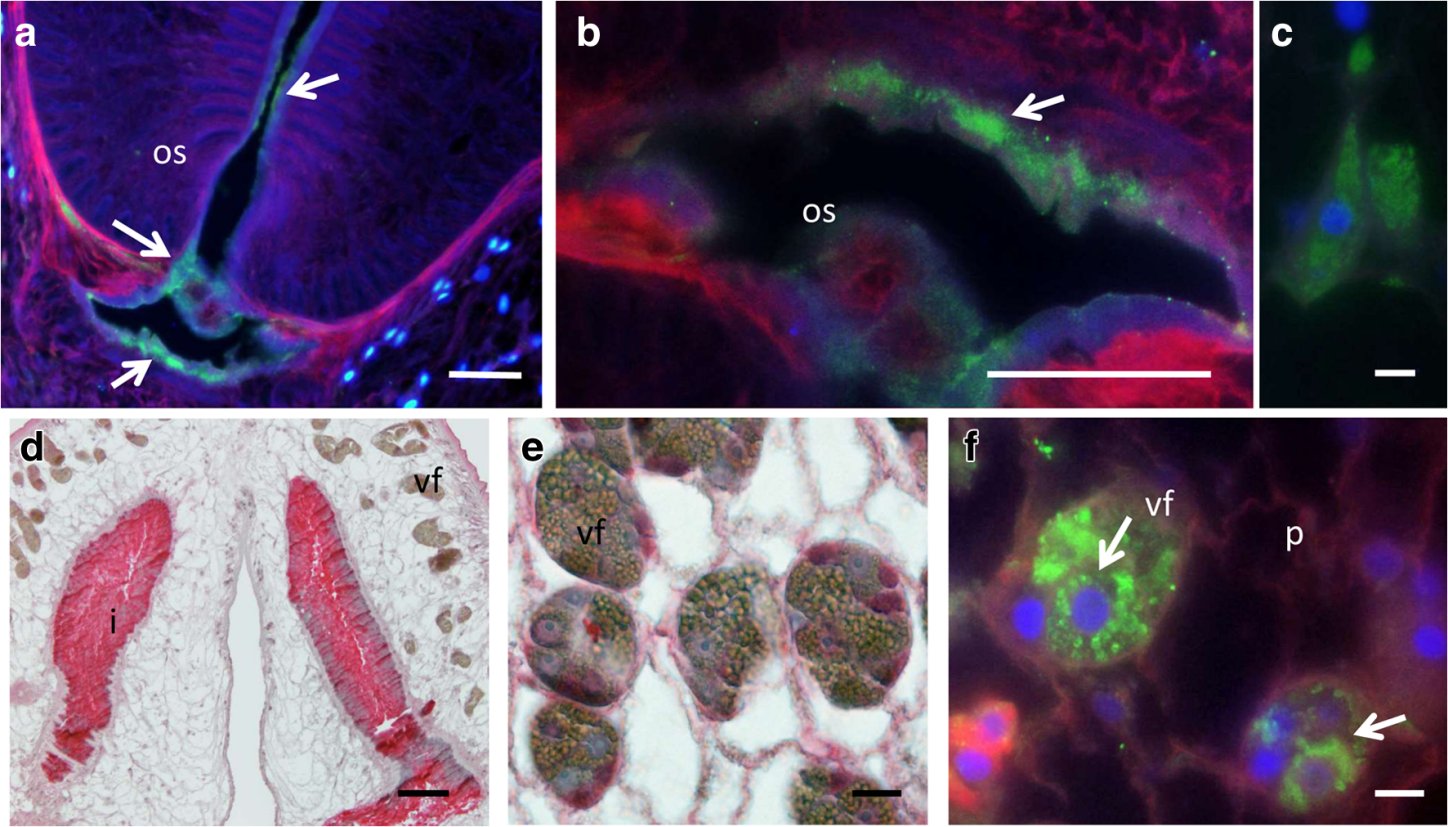
More detailed immunohistological localization studies of CP-6 in adult *P. kellicotti* worms by immunofluorescence (**a**–**c**, **f**) or APAAP (**d**, **e**). **a**, **b** Intense green labeling of the mouth and the outer area of the oral sucker. **c** Distinct labeling of the cytosol of a cell close to the mouth. **d** Intense staining of the intestine that exceeds the alkaline phosphatase background pattern in the intestine (compare Fig. 1**a**, **b**). **e**, **f** Intense granular staining of the vitelline follicles. Os, oral sucker; vf, vitelline follicle; p, parenchyma. Scale bar for **a**, **b**, **d** 100 μm and for **c**, **f** 10 μm

CP-6 is mainly localized in the tegument at the parasite-host interface (Fig. 2g). Some staining was detected in the vitelline follicles, and strong staining was seen in the full thickness of the intestine (not limited to the inner lining) (Figs. 2c, 4d–f). Staining was also present in the oral and ventral suckers (Figs. 3c, d; 4a–c). CYS-2 was localized to the tegument, vitelline follicles, and intrauterine eggs (Fig. 2i, j), while antibodies to MYO-1 bound strongly to the tegument. Weaker MYO-1 staining was observed in the parenchyma and in intrauterine eggs (Fig. 2k, l).

**Fig. 5:**
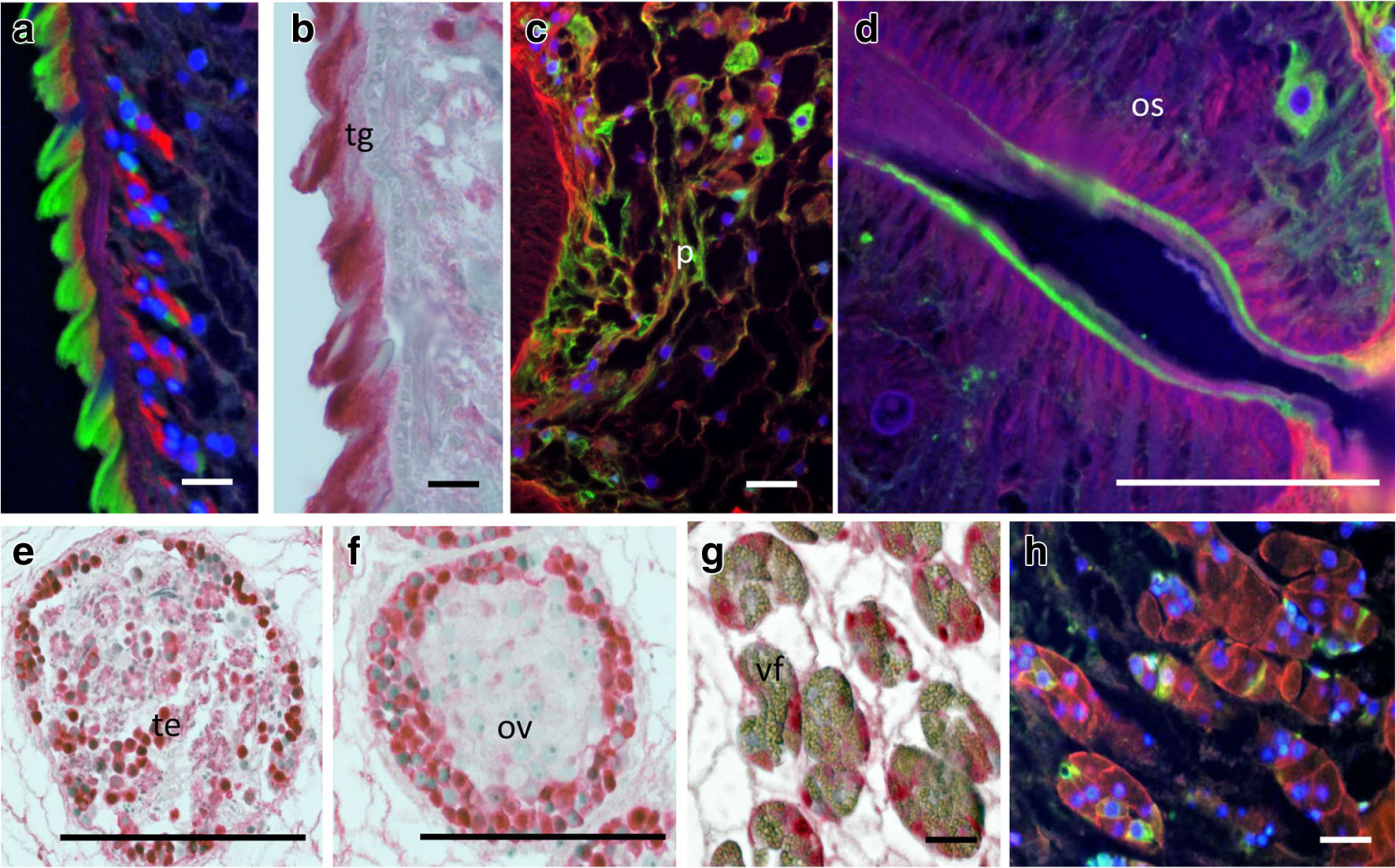
More detailed immunohistological localization of MDP in adult *P. kellicotti* flukes using the immunofluorescence (**a**, **c**, **d**, **h**) or APAAP (**b**, **e**–**g**). **a**, **b** Intense labeling of outer tegument. **c** Labeling of the parenchyma in vicinity of the oral sucker. **d** Intense staining of outer parts of the oral sucker. **e**, **f** Intense granular staining of testis and ovary. **g**, **h** Granular staining of single cells within the vitelline follicle. Os, oral sucker; vf, vitelline follicle; p, parenchyma; tg, tegument; os, oral sucker; te, testis; ov, ovary. Scale bar for **a**, **b**, **c** 10 μm and for **d**, **e**, **f** 10 μm

Staining for MDP was widespread and it appears that this protein is abundant in many worm tissues (see Supplemental Figure 1). MDP staining was especially intense in the tegument, inner parts of the suckers, the intestine, and the parenchyma (Figs. 2d, m; 3e, f; 5). Staining was also present in the testis, ovaries, and the vitelline follicles (Fig. 4). Analysis of total adult *P. kellicotti* extract by Western blot using this antibody supports the finding that this is an abundant protein and that the antibody detects specifically only a single protein band at around 8.4 kDa.

Localization studies showed that EYF was abundant in the vitelline follicles and intrauterine eggs (Fig. 2o, p), but not in other tissues of *P. kellicotti*. These results show that immunolocalization is not only helpful for a better characterization of proteins of interest, but it also helps to experimentally identify proteins that are secreted and/or localized at the parasite-host interface, and maybe exposed to the human immune system and, therefore, may represent preferred targets for serodiagnostics.

### Evaluation of recombinant proteins as serodiagnostic antigens

The diagnostic potential of the five antigen candidates was assessed by Western blot with a panel of sera from 17 subjects with proven *Paragonimus* infections (13 *P. kellicotti* cases from MO and 4 *P. westermani* cases from the Philippines) and with sera from *Paragonimus*-negative controls with or without other trematode infections (Table 3). In this pilot evaluation, CP-6 and MYO-1 had a sensitivity of 100% with sera from paragonimiasis patients vs sensitivities of 76, 41, and 24% for MDP, EYF, and CYS-2, respectively. All clinical cases had antibodies reactive to antigens present in total adult worm extract. Some of the infected subjects had detectable eggs in sputum or stool. In total, 7 *P. kellicotti* and 4 *P. westermani* infected subjects had detectable eggs, and of those, 6 *P. kellicotti* and 1 *P. westermani* samples had antibodies reactive with recombinant EYF by Western blot. None of the 28 control sera from persons with no infection or with infections with other helminth parasites had antibodies to Myo-1. MDP, EYF, and CYS-2 also appeared to have good specificity, but fewer negative control sera were tested with these antigens because of their sub-optimal sensitivity.

**Table 3:** Results of the pilot evaluation of the serodiagnostic potential of five recombinant *P. kellicotti* proteins, cysteine protease-6 (CP-6), cystatin-2 (CYS-2), myoglobin-1 (MYO-1), a microbial defense protein (MDP), and an egg yolk ferritin (EYF). For all proteins, total IgG antibodies were detected by Western blot, but for Cp-6 antibodies of the IgG4, subclass was also assessed in order to increase specificity.

Sera type	Cp-6		Cys-2	Myo-1	Mdp	Eyf
	IgG, pos/N	IgG4, pos/N	IgG, pos/N	IgG, pos/N	IgG, pos/N	IgG, pos/N
*P. kellicotti*	13/13	13/13	4/13	13/13	10/13	6/13
*P. westermani*	4/4	4/4	0/4	4/4	3/4	1/4
Other helminth infections	5/5	0/23	0/5	0/23	0/5	0/5
US non-infected	1/1	0/5	0/2	0/5	0/2	0/2

**Fig 6:**
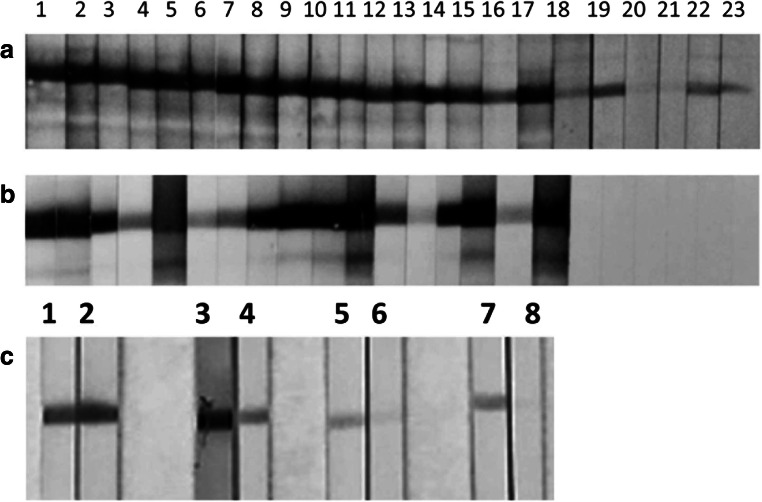
Western blot detection of antibodies to rCP-6 in patient and control sera. **a** Detection of total IgG antibodies. **b** Detection of IgG_4_ subclass antibodies. Lanes 1–10 and 12–14 sera are from individuals with *P. kellicotti* infection; lanes 11, 15–17 sera are from individuals with *P. westermani* infection; lanes 18–23, tested sera were from individuals with *Fasciola hepatica* or *Schistosoma mansoni* infections. **c** Western blot analysis of selected patient sera before and after successful treatment using rCP-6 as antigen. Lanes 1, 3, 5, and 7 before treatment and lanes 2, 4, 6, 8, 53 days, 224 days, 42 days, and 59 days after treatment

All sera from paragonimiasis patients contained IgG antibodies to CP-6, but six control sera also had antibodies that reactivated with the antigen (Fig. 6a). Specificity improved to 100% with no loss of sensitivity when we used anti-human IgG4 as the secondary antibody in CP-6 (Table 3; Fig. 6b).

We performed CP-6 IgG4 Western blots with paired serum samples from four patients with *P. kellicotti* infections that were collected before and after they were successfully treated with praziquantel. Treatment success was defined as resolution of clinical symptoms associated with infection. One patient’s sera had similar band intensities before and 53 days post-treatment. However, band intensities decreased 224, 42, and 59 days after treatment in three other patients (Fig. 6c). These results suggest that although IgG4 antibody titers to CP-6 tend to decrease after successful treatment, they sometimes remain detectable for some months.

## Discussion

Human paragonimiasis in an important FBT infection, and it was estimated that in 2017, this group of NTDs is responsible for 1,870,700 years lived with disabilities (YLD) (GBD 2017 Disease and Injury Incidence and Prevalence Collaborators 2018). Among the FBT infections, about 50% of the YLD are assumed to be caused by paragonimiasis (Furst et al. 2012). However, these estimates do not include infections in Africa or North America, and because of underdiagnosis and underreporting, the global number of infections is likely to be higher. Furthermore, pathogenicity and the ascribed disability weight for paragonimiasis is species-dependent and may also be an underestimate (Feng et al. 2018). Therefore, improved diagnosis for paragonimiasis would be important not only for clinical care, but also for a more accurate estimation of its public health importance.

We amplified, expressed, characterized, and evaluated five recombinant *P. kellicotti* proteins for their potential as serodiagnostic antigens. Two proteins, CYP-6 and MYO-1, show great potential as serodiagnostic antigens for paragonimiasis. Immunolocalization studies showed that four of the five candidate diagnostic antigens were present in the tegument of adult worms, at the host-parasite interface. Protein EYF was only detected in the vitellaria and the eggs, and it is possible that antibodies to this protein only develop after worms have matured to adulthood with egg production.

Few proteins have been localized in adult *Paragonimus* flukes before, and our thorough immunolocalization of five proteins with antibodies raised against recombinant proteins has provided significant new information. MYO-1 was previously localized in *P. westermani*, and our data confirm those findings with strong staining throughout the parenchymal tissue and the tegument, and absent or weak staining of the embryonic cells inside the eggs (de Guzman et al. 2007). There are many different cysteine proteases in *Paragonimus*, and their tissue localization appears to vary. Prior studies have localized them in the intestinal epithelium (Choi et al. 2006), esophagus (Yoonuan et al. 2016), and the tegument in the anterior part of the fluke including the oral sucker (Na et al. 2006).

Based on recently generated genomic information, all five of the proteins selected for this study showed a high degree of conservation between four *Paragonimus* species (three from Asia and one from North America). Although MYO-1 of *P. kellicotti* is 90–95% identical to the orthologs in the genomes of the other three *Paragonimus* species, it is only 53% and 58% identical to orthologs in the genomes of the related trematodes *Clonorchis sinensis* and *O. viverrini*, respectively (Supplemental Table S2) (Wang et al. 2011; Young et al. 2014). Similarly, CP-6 of *P. kellicotti* is 79–86% identical to orthologs in the genomes of the other three *Paragonimus* species, but only 49% and 55% identical to the closest orthologs in the genomes of the related trematodes *C. sinensis* and *O. viverrini*, respectively (Supplemental Table S2). Even lower degrees of similarities were observed for other trematode species such as *Fasciola gigantica*, *Fasciola hepatica*, and *Schistosoma mansoni*. Therefore, the relatively high genus-specific conservation in *Paragonimus* may facilitate the development of pan-*Paragonimus* serology tests.

Few *Paragonimus* proteins have been evaluated as potential serodiagnostic antigens, and most have been evaluated with sera from persons infected with a single *Paragonimus* species. The EYF antigen of *P. westermani* was reported to have high specificity (Kim et al. 2002b). However, in experimentally infected cats, it took 13 weeks following infection before antibodies were detectable by Western blot. Our study confirmed the high specificity of this antigen, but showed unsatisfactory sensitivity, especially for sera from persons with early infections. Myoglobins are highly abundant proteins in adult *P. westermani* and *P. kellicotti* (de Guzman et al. 2007; McNulty et al. 2014), but they have not been previously studied as diagnostic antigens for paragonimiasis. While cystatins have not been tested for their direct serodiagnostic potential, a cystatin capture assay has been used to capture cysteine proteinases as diagnostic antigens (Ikeda 1998).

Cysteine proteases have been suggested as targets for serodiagnosis of paragonimiasis (Blair et al. 2016). Cysteine protease fractions have been partially isolated from excretory/secretory products from *P. westermani*, and this antigen preparation showed increased specificity compared to a total worm extract (Ikeda et al. 1996). *Paragonimus* species have many cysteine proteases, and only a few of them have been examined as diagnostic antigens. For example, eleven cathepsin F family cysteine proteinases of *P. westermani* were reported to have diagnostic sensitivities that ranged between 38.4 and 84.5% with specificities above 87% (Ahn et al. 2015). We have previously reported that, although cysteine proteases are not among the most abundant proteins in adult *P. kellicotti* worms, three of them are among the 25 most immunoreactive proteins in adult worm extracts. Indeed, CP-6 was the most abundantly detected immunoreactive protein based on spectral counts (McNulty et al. 2014). The present study showed that a Western blot for IgG4 antibodies to *P. kellicotti* CP-6 had high sensitivity and specificity, but the parallel IgG assay had low specificity. A previous study with *P. westermani* CP-6 (Western blot for total IgG) reported a sensitivity of 77.5% and a specificity of 96.7% (Ahn et al. 2015).

Previous studies showed that Western blot assays with crude *P. westermani* or *P. kellicotti* adult worm extracts can be useful for serodiagnosis of paragonimiasis (Fischer et al. 2013; Slemenda et al. 1988). The present study shows that *P. kellicotti* rCP-6 and rMYO-1 can be used in place of native parasite antigen extracts. This may help to standardize the serological diagnosis of paragonimiasis and make testing more widely available. Recombinant CYP-6 and MYO-1 are very promising as serodiagnostic antigens that appear to have excellent sensitivity and specificity when IgG4 (CP-6) or total IgG (MYO-1) antibodies are detected. However, this pilot evaluation only included a limited panel of samples, and larger numbers of samples from different endemic areas will need to be tested to fully evaluate the diagnostic potential of these antigens.

## Supplementary information

Supplemental Fig. S1. Overview of immunohistological localization of MDP in adult *P. kellicotti* flukes using APAAP. Intense staining is seen in most parts of the fluke with exception of the muscles and eggs. Os, oral sucker; vs, ventral sucker; vf, vitelline follicle; p, parenchyma; ut, uterus, te, testis. Scale bar 1 mm. (PNG 3929 kb)

High resolution image (TIF 1030 kb)

ESM 2 (PDF 353 kb)
