# Supplementary figures and images for: Characterization and localization of antigens for serodiagnosis of human paragonimiasis
Source: Parasitol Res. 2021 Jan 8;120(2):535–45. doi: 10.1007/s00436-020-06990-z (PMC7854406; doi:10.1007/s00436-020-06990-z)

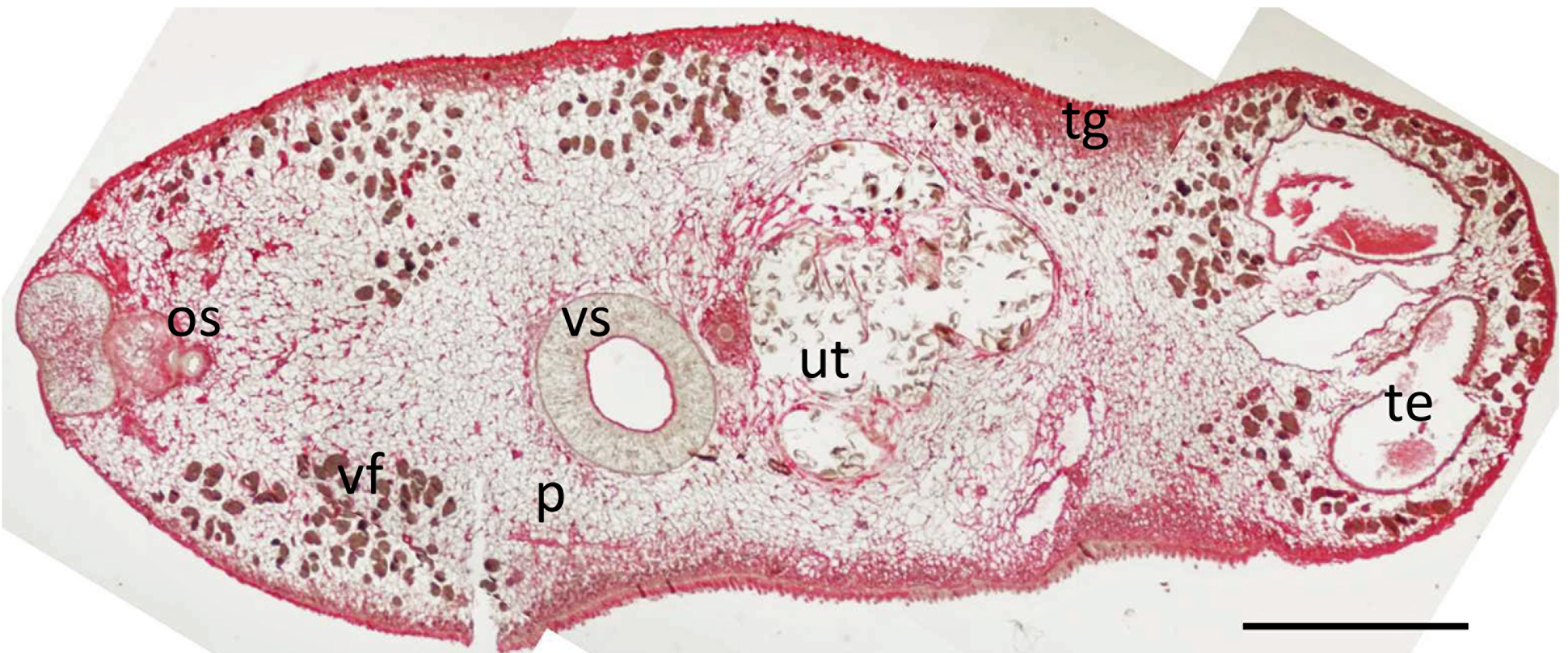

Supplement: Supplementary file 1 — Overview of immunohistological localization of MDP in adult P. kellicotti flukes using APAAP. Intense staining is seen in most parts of the fluke with exception of the muscles and eggs. Os, oral sucker; vs, ventral sucker; vf, vitelline follicle; p, parenchyma; ut, uterus, te, testis. Scale bar 1 mm. (PNG 3929 kb) [file 436_2020_6990_Fig7_ESM.png]
