# Supplementary material for: Characterization and localization of antigens for serodiagnosis of human paragonimiasis
Source: Parasitol Res. 2021 Jan 8;120(2):535–45. doi: 10.1007/s00436-020-06990-z (PMC7854406; doi:10.1007/s00436-020-06990-z)
Supplement: Supplementary file 3 — (PDF 353 kb) [file 436_2020_6990_MOESM2_ESM.pdf]

Supplemental Table S1. Sequences of primers used to amplify and express antigen candidates from *P. kellicotti* cDNA.

Clone designation	Transcript designation	Primer designation, sequence 3'-5'
Cp-6	Pk00527	*PK00527CACC,* caccCTGCCTACCGTCAGTTGTCTTACTTTCTTGC<br>*PK00527TAG,* ctaACGGATAATTGCAGACGAAACAAGTTTTTCG
Cys-2	Pk140546	*Pk140546CACC,* caccTTTAGATACACATCTTGTCTGTTATTCATCATG<br>*Pk140546TAG,* ctaAAAATATACCAAGGGGTCAGCGTGAGTTTTGTTGCG
Myo-1	Pk120808	*PK120808CACC,* caccATGGCGCCACTCACACAGACTGAAGTCGACG<br>*PK120808TAG,* ctaAGCAATCACGGGAAAAGCGTGTTTGAGGAAC
Mdp	Pk131774	*Pk131774CACC,* caccTACACCGAAGCTCGTCCGGATTCGC<br>*Pk131774TAG,* ctaGTTCTCGACATATTTCTCAATTCGGTCAG
Eyf	Pk145008	*Pk145008CACC,* caccATGCCCGGGTTCTGCAAGTTCTTCCGTCTATGC<br>*Pk145008TAG,* ctaAAAAGGATAGATGAATGCAGCTATACCAGC

Supplemental Table S2. Comparison of the amino acid sequences of the *P. kellicotti* CP-6 and MYO-1 serodiagnostic antigen candidates with the closest orthologue in the genomes of the trematodes *Clonorchis sinensis*, *Fasciola gigantica*, *Fasciola hepatica*, *Fasciolopsis buski*, *Opisthorchis viverrini, Schistosoma mansoni* and *Echinostoma caproni*.

	P. kellicotti CP-6				*P. kellicotti* MYO-1			
Species	prot_name	% similarity	Align length	E-value	prot_name	% similarity	Align length	E-value
C. sinensis	CLF_100357	49	335	8.21E-106	CLF_111027	53	149	2.73E-59
F. gigantica	FGIG_05822	47	241	2.00E-64	FGIG_04911	52	149	9.94E-52
F. hepatica	D915_006264	44	321	1.24E-75	D915_003344	50	149	1.32E-50
F. buski	FBUS_01115	49	309	1.00E-100	FBUS_03031	53	146	2.00E-52
O. viverrini	T265_12433	55	315	2.00E-122	T265_08533	58	146	2.00E-61
S. mansoni	XP_018649822	46	321	5.53E-92	Smp_162360	42	149	7.01E-38
E. caproni	ECPE_LOCUS6911	51	301	1.00E-111	ECPE_LOCUS2838	50	146	3.00E-47
